# Factors that predict early treatment failure for patients with locally advanced (T4) breast cancer

**DOI:** 10.1038/sj.bjc.6604384

**Published:** 2008-05-27

**Authors:** E Montagna, V Bagnardi, N Rotmensz, J Rodriguez, P Veronesi, A Luini, M Intra, E Scarano, A Cardillo, R Torrisi, G Viale, A Goldhirsch, M Colleoni

**Affiliations:** 1 Research Unit in Medical Senology, Department of Medicine, European Institute of Oncology, Milan, Italy; 2 Division of Epidemiology and Biostatistics, European Institute of Oncology, Milan, Italy; 3 Department of Statistics, University of Milan-Bicocca, Milan, Italy; 4 Frontier Science and Technology Research Foundation, Southern Europe, Milan, Italy; 5 Division of Senology, European Institute of Oncology, Milan, Italy; 6 University of Milan School of Medicine, Milan, Italy; 7 Division of Pathology, European Institute of Oncology, Milan, Italy; 8 Department of Medicine, European Institute of Oncology, Milan, Italy

**Keywords:** prognostic factors, preoperative therapy, surgery, locally advanced breast cancer

## Abstract

Locally advanced breast cancer (LABC) is associated with dire prognosis despite progress in multimodal treatments. We evaluated several clinical and pathological features of patients with either noninflammatory (NIBC, cT4a-c) or inflammatory (IBC, cT4d) breast cancer to identify subset groups of patients with high risk of early treatment failure. Clinical and pathological features of 248 patients with LABC, who were treated with multimodality treatments including neoadjuvant chemotherapy followed by radical surgery and radiotherapy were reassessed. Tumour samples obtained at surgery were evaluated using standard immunohistochemical methods. Overall, 141 patients (57%) presented with NIBC (cT4a-c, N0-2, M0) and 107 patients (43%) with IBC (cT4d, N0-2, M0). Median follow-up time was 27.5 months (range: 1.6–87.8). No significant difference in terms of recurrence-free survival (RFS) (*P*=0.72), disease-free survival (DFS) (*P*=0.98) and overall survival (OS) (*P*=0.35) was observed between NIBC and IBC. At the multivariate analysis, patients with ER- and PgR-negative diseases had a significantly worse RFS than patients with ER- and/or PgR-positive diseases (hazard ratio: 2.47, 95% CI: 1.33–4.59 for overall). The worst RFS was observed for the subgroup of patients with endocrine nonresponsive and HER2-negative breast cancer (2-year RFS: 57% in NIBC and 57% in IBC) A high Ki-67 labelling index (>20% of the invasive tumour cells) and the presence of peritumoral vascular invasion (PVI) significantly correlated with poorer RFS in overall (HR 2.69, 95% CI: 1.61–4.50 for Ki-67>20% and HR 2.27, 95% CI: 1.42–3.62 for PVI). Patients with endocrine nonresponsive LABC had the most dire treatment outcome. High degree of Ki-67 staining and presence of PVI were also indicators of higher risk of early relapse. These factors should be considered in therapeutic algorithms for LABC.

Locally advanced breast cancer (LABC) is a heterogeneous group of diseases. It include tumours with locoregional lymph node metastases, primary breast carcinomas infiltrating skin or chest wall (cT4 a-c), as well as inflammatory breast carcinoma (cT4d, IBC), the latter commonly considered as a distinct clinicopathological entity (Singletary *et al*, 2002). The American Joint Committee on Cancer (AJCC) based the IBC diagnosis on the first clinicopathological description by Haagensen (1971). Typically, the clinical presentation of IBC includes tenderness, induration, warmth, peau d’orange, often without an underlying palpable mass; these signs progress quite rapidly (Greene *et al*, 2002; Anderson *et al*, 2003). Several retrospective studies that compared patients with primary IBC and those with noninflammatory breast cancer (NIBC) led to the notion that IBC is clinically and biologically more aggressive (Low *et al*, 2004; Cristofanilli *et al*, 2007). Inflammatory breast cancer more frequently than NIBC is characterised by an early age at diagnosis, negative hormone receptor status, high nuclear grade, HER2/neu gene amplification and overexpression, and by pronounced neo-angiogenic and invasive features (Turpin *et al*, 2002; Anderson *et al*, 2003; Sawaki *et al*, 2006).

In contrast, controversies exist on the prognostic value of the T4 category for NIBC according to the TNM classification. Indeed, several authors suggested that NIBC currently classified as T4a–c should be classified according to tumour size, because the size of the primary tumour or the nodal involvement, rather than direct invasion of the chest wall or ulceration of the skin, might better indicate prognosis (Gueth *et al*, 2007).

Limited information on prognostic and predictive parameters for both IBC and NIBC is currently available, and it is largely derived from old retrospective series, collected during several years. In fact, despite possible differences in the clinical and biological behaviour of a T4 presentation, either inflammatory or not, patients with either presentation are commonly treated with similar combined-modality regimens.

To seek information on the prognostic value of clinical and pathological features of both IBC and NIBC collected at surgery and to identify a subset of patients with very high risk of early relapse, we evaluated the treatment outcome in 248 patients with LABC (cT4a–d) who were operated at the European Institute of Oncology (EIO) between 1999 and 2006.

## PATIENTS AND METHODS

### Patients

Prospectively collected data from consecutive patients with histologically and/or cytologically proven clinical (c) stage T4a–d, N0-2 and submitted to surgery at the IEO from November 1999 to November 2006 were analysed. Clinical diagnosis of T4 was performed by the surgeon who asked an oncologist consult for the patients to start neoadjuvant treatment. In agreement with T4d classification in the AJCC system, clinical diagnosis of IBC required presence of erythema, heat, ridging or peau d’orange on breast. Data on the patient's medical history, concurrent diseases, type of surgery, histopathological features and results of staging procedures (blood chemistry, haematological values, bone scan, chest film and upper abdominal ultrasound examination) were reviewed.

### Treatment

Patients were treated under best-known multidisciplinary management. All patients received preoperative chemotherapy (plus/minus endocrine therapy) and adequate local treatment. In all, 106 patients with IBC underwent a modified radical mastectomy followed by external beam radiotherapy. Owing to the presence of skin involvement at the onset of diagnosis, an extensive skin removal was performed and in selected cases a musculocutaneous flap was needed for surgical closure. Immediate breast reconstruction using an expander or a prothesis was not used because of the need for postoperative locoregional radiotherapy. Postoperative irradiation was proposed to all patients. The selection of adjuvant systemic treatment was based upon indicators of responsiveness to treatment (endocrine responsiveness of the tumour) and evaluation of risk. For patients with endocrine responsive disease, adjuvant endocrine therapy alone according to menopausal status was prescribed (tamoxifen or aromatase inhibitor) for a duration of 5 years in postmenopausal patients and the combination of tamoxifen for 5 years plus gonadotropin releasing hormone analogues for at least 2 years in premenopausal patients (Goldhirsch *et al*, 1998). In patients at higher risk (e.g., pN1a disease) and/or features of limited endocrine responsiveness, chemotherapy was added to the endocrine treatment programme.

### Response criteria

Responses to neoadjuvant chemotherapy were evaluated according to both radiological (breast ultrasound plus Rx mammography) and clinical evaluation and graded according to standard WHO criteria. Pathological complete remissions (pCRs) were evaluated according to the criteria by Kuerer *et al* (1999). In particular, the absence of invasive cancer on both the primary breast tumour and axillary lymph nodes qualified for pCR.

### Pathology and immunohistochemistry

This is a single institution study. All patients had pathological evaluation performed during final surgery at the EIO. Surgical specimens were extensively sampled for the evaluation of residual tumour after primary chemotherapy. In case of lack of gross evidence of tumour, the quadrantectomy specimens were entirely blocked in paraffin and examined histologically, as were the tumour-bearing quadrants of the mastectomies. In the latter cases, the other quadrants were also thoroughly evaluated with the examination of at least three tissue blocks.

Immunostaining experiments for the localisation of ER and PgR, HER2 protein and Ki-67 antigen were performed on consecutive tissue sections from the residual tumour after surgery, as previously reported (Colleoni *et al*, 1999). The following primary antibodies were used: the monoclonal antibody (MAb) to ER (Dako, Glostrup, Denmark; at 1/100 dilution), the MAb to PgR (Dako; 1/800), the MIB-1 MAb to the Ki-67 antigen (Immunotech, Marseille, France; 1/1200) and the polyclonal antiserum (Dako; 1/3200) to the HER2 protein.

Only nuclear reactivity was taken into account for ER, PgR and Ki-67 antigen, whereas only an intense and complete membrane staining in >10% of the tumour cells qualified for HER2 overexpression (3+). The results were recorded as the percentage of immunoreactive cells over at least 2000 neoplastic cells. The value of 20% for Ki-67 labelling index (LI) was used as a cutoff in distinguishing tumours with low (<20%) and high (⩾20%) proliferative fraction (Colleoni *et al*, 1999). Steroid hormone receptors status was classified as negative (lack of any ER and PgR immunoreactivity, or <1% immunoreactive tumour cells), low (ER and/or PgR <10% immunoreactive tumour cells) or positive (ER and/or PgR ⩾1% of the cells).

### Statistical analysis

Characteristics of the two disease groups (NIBC and IBC) were compared using Pearson's *χ*^2^ test. The primary end point was recurrence-free survival (RFS). Recurrence-free survival was measured from the date of surgery to any locoregional invasive recurrence (including ipsilateral breast recurrence), distant recurrence, death for breast cancer or date of last follow-up visit, whichever occurred first. Patients who died for nonbreast cancer cause or patients who experienced a second primary cancer (including contralateral breast cancer) were considered censored at their event date. Second end points were disease-free survival (DFS) and overall survival (OS). Disease-free survival was defined as the length of time from the date of surgery to any relapse (including ipsilateral breast recurrence), the appearance of a second primary cancer (including contralateral breast cancer), death or the date of last follow-up visit, whichever occurred first. Overall survival was determined as the time from surgery until the date of death (from any cause) or the date of last contact. The median time of follow-up was calculated as both the median observation time among all patients and the median observation time among patients still alive at their last follow-up. Plots of the survival curves were drawn using the Kaplan–Meier method. The log-rank test was used to assess the survival difference between the two disease groups and, separately for each group, between patient and tumour characteristics at surgery. Patients achieving a complete pathological response were excluded in the analyses evaluating histopathological characteristics of the tumour assessed at surgery (two patients in NIBC and five patients in IBC). Cox's proportional hazard regression models were used to assess the prognostic significance of clinical and histopathological characteristics of the tumour on RFS and to estimate the association between disease group and RFS, after statistical adjustment for patient and disease characteristics.

The model included year of surgery, age at surgery, number of positive lymph nodes, hormone receptor status, HER2 overexpression, Ki-67 LI, peritumoral vascular invasion (PVI) and pathological response. In the univariate analysis, the Wald test was used to evaluate significance of individual coefficients, and the likelihood ratio test was used to assess factor with more than two levels (e.g., lymph node involvement). Factors that were significant at a *P*-value of 0.10 or less were entered into the multiple regression model. Results from Cox's models were expressed as hazard ratios (HRs) with 95% confidence intervals (CIs). All analyses were performed with the SAS software version 8.02 (SAS, Cary, NC, USA). All *P*-values were two sided.

## RESULTS

### Results

Overall, 504 patients were identified with a cT4 disease and were available for the analysis. Patients who presented with recurrent tumours (*n*=19), metastatic disease at presentation (*n*=99), other previous tumour (*n*=18), no primary chemotherapy (*n*=84) and male breast cancer (*n*=5) were excluded. Finally, we removed patients with LABC who received trastuzumab therapy (31 patients), and the current analysis was restricted to 248 patients.

Patients’ characteristics are shown in Table 1. Tumours were classified as IBC for 107 patients (43%) and as NIBC for 141 (57%). There were no significant differences between the characteristics of the two groups, except for the increased frequency of IBC in recent years. As expected, the prevalent tumour histotype in both groups was invasive ductal carcinoma, NOS (not otherwise specified). At final surgery, the majority of patients presented with >4 positive axillary nodes (70.7% in NIBC and 72.2% in IBC). An elevated percentage of staining for Ki-67 was observed in 64 patients (48.9%) with NIBC and in 56 patients (57.7%) with IBC. HER2 was overexpressed in 25.4 and 22.5% of NIBC and IBC, respectively.

### Treatment

Primary treatments are detailed in Table 2. All patients received primary chemotherapy: infusional regimens were administered to 54 patients with NIBC and to 61 patients with IBC. A total of 35.4 and 22.4% of patients with NIBC and IBC received a taxane and anthracycline-containing chemotherapy. A total of 52.5 and 72.0% of patients with NIBC and IBC, respectively, received an anthracycline combination chemotherapy. One hundred and twenty-eight (90.8%) patients with NIBC and 106 (99.1%) patients with IBC had a total mastectomy as the surgical treatment. Thirteen patients with NIBC (9.2%) underwent quadrantectomy with axillary dissection followed by external beam radiotherapy.

Adjuvant radiotherapy was performed in a similar proportion of patients of the two cohorts: NIBC (94.3%), IBC (94.4%). Adjuvant chemotherapy (with or without addition of endocrine therapy) was prescribed to 81 (57.4%) and 57 (53.3%) patients with NIBC and IBC, respectively. There was no significant difference in terms of response to preoperative chemotherapy between the two groups (partial response: 56.7% for NIBC and 57.9% for IBC).

### Events

By 23 October 2007, the median follow-up among all patients was 27.5 months (range: 1.6–87.8), and median follow-up among patients still alive at their last follow-up was 31.0 months (range: 2.0–87.8). A total of 25 out of 248 patients were lost to follow-up.

**Figure 1:**
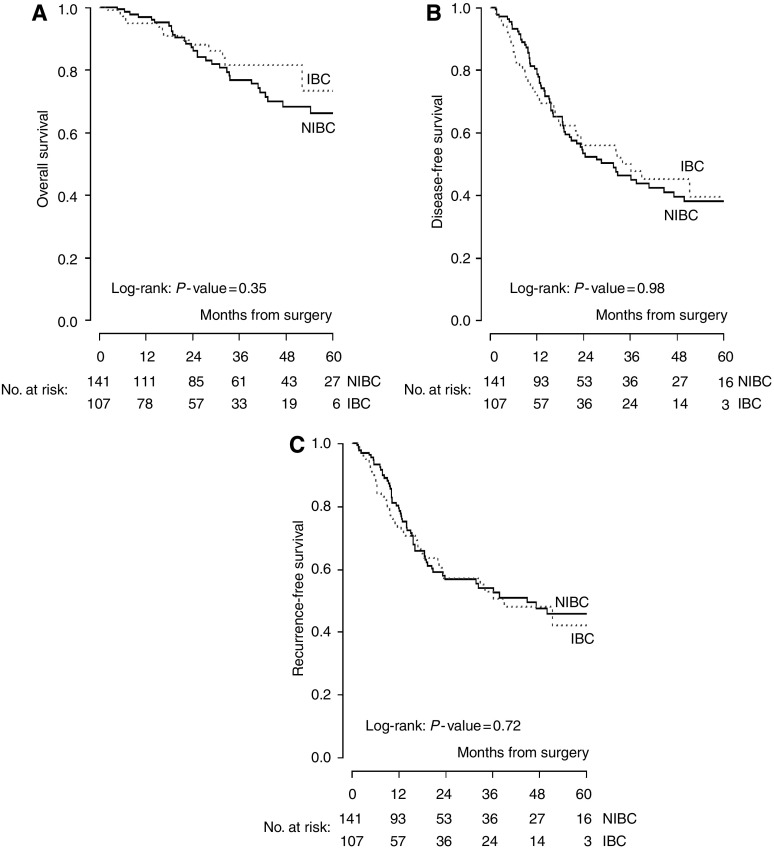
(**A**) Overall survival, (**B**) DFS and (**C**) RFS in IBC and NIBC.

The Kaplan–Meier curves for OS, DFS and RFS are displayed in Figure 1A–C. The estimated proportion of surviving patients at 2 and 5 years was 86% (95% CI: 79–93%) and 66% (95% CI: 56–77%) for patients with NIBC and 88% (95% CI: 81–95%) and 73% (95% CI: 56–90%) for IBC patients.

Recurrence-free survival at 2 years was 57% (95% CI: 47–66%) and 57% (95% CI: 46–68%) for NIBC and IBC. No significant difference in terms of RFS (*P*=0.72), DFS (*P*=0.98) and OS (*P*=0.35) was observed between the two groups.

**Figure 2:**
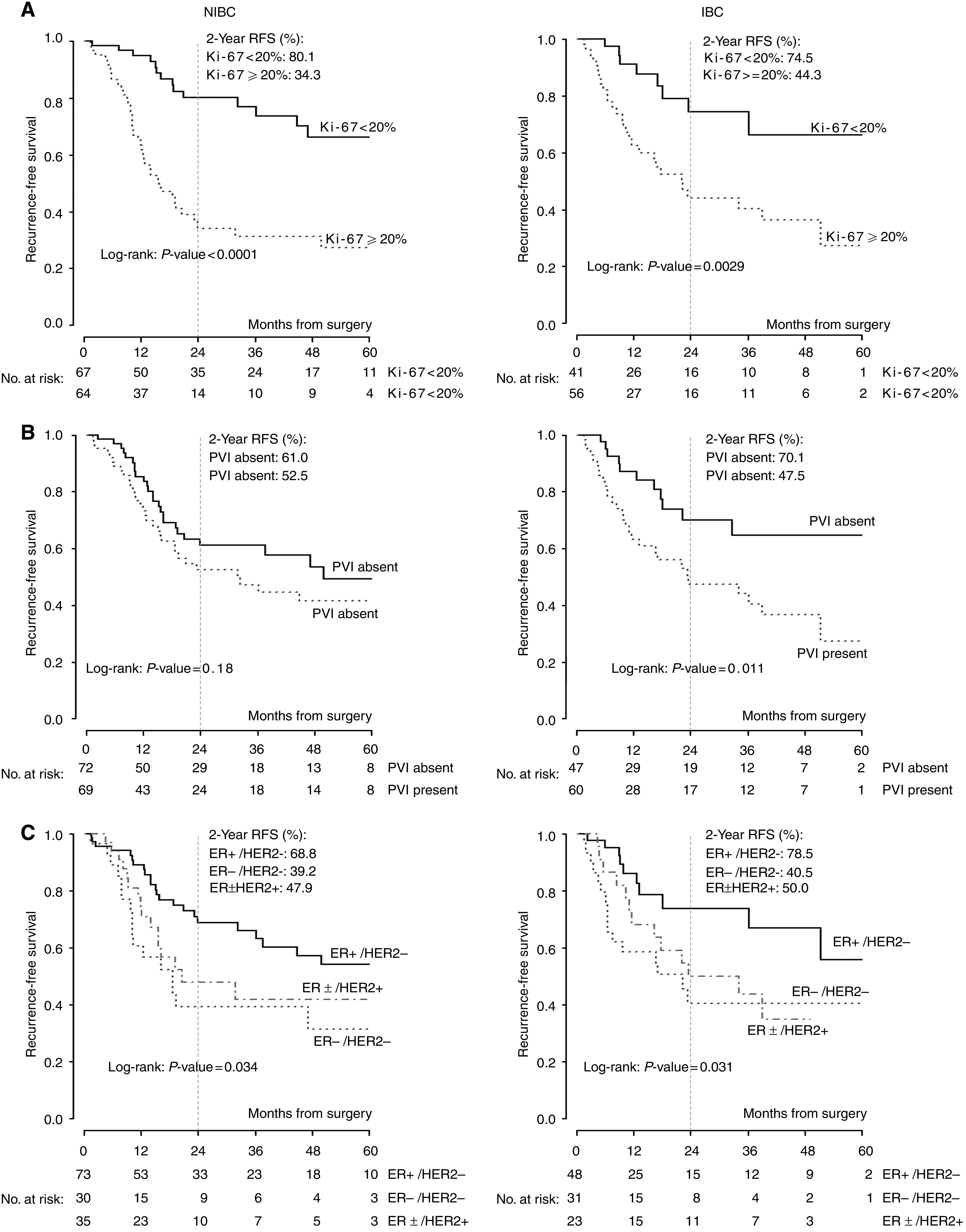
Recurrence-free survival, stratified by (**A**) Ki-67, (**B**) PVI and (**C**) ER/HER2 status in NIBC and IBC.

High degree of immunostaining for Ki-67 correlated with worse RFS in NIBC (*P*<0.0001) and in IBC (*P*=0.0029) at the univariate analysis as displayed in Figure 2A. Presence of PVI was associated with poor RFS in NIBC (*P*=0.18) and IBC (*P*=0.01) as shown in Figure 2B.

An exploratory analysis showed worse RFS for subgroup of patients with basal-like (HER2 negative and ER negative) compared with luminal (ER positive and HER negative) and HER2-positive breast cancer. Recurrence-free survival at 2 years for ER- and HER2-negative breast cancer was 39.2% in the NIBC group and 40.5% in IBC patients as displayed in Figure 2C.

### Multivariate analysis

Independent association between biological features and probability of relapse was analysed, and the results are displayed in Tables 1, 2 and 3.

Patients with ER- and PgR-negative disease had a significantly shorter RFS than patients with ER- and/or PgR-positive disease (HR 2.47, 95% CI: 1.33–4.59, *P*=<0.001). A high proportion of Ki-67 cell staining and presence of PVI significantly correlated with poorer RFS (HR 2.69, 95% CI: 1.61–4.50, *P*=<0.001 for Ki-67>20% and HR 2.27, 95% CI: 1.42–3.62, *P*=<0.001 for PVI).

The lymph nodes involvement, years of surgery, age of patients, HER2 status, neoadjuvant response and chemotherapeutic regimen did not correlate at multivariate analysis with poorer RFS. When we included in the analysis patients who received trastuzumab therapy, HER2 status also did not correlate with worse RFS.

## DISCUSSION

One of the most important factors predicting the risk of relapse in patients with LABC after radical surgery is the type of presentation. In particular, as recently reported on a large retrospective series, the inflammatory presentation was associated with significantly worse RFS and OS when compared with a noninflammatory locally advanced disease (NIBC) (Cristofanilli *et al*, 2007). Unfortunately, a limited number of prognostic and predictive factors were looked at within the subgroups with either inflammatory (IBC) or NIBC. These included number of involved lymph node (Klauber-DeMore *et al*, 2006), tumour size (Carter *et al*, 1989), hormone receptor status (Huang *et al*, 2005) and indicators of tumour proliferation (Aas *et al*, 2003).

Current knowledge on these prognostic factors is largely dependent upon older retrospective series. Surgical and staging procedures, neoadjuvant and adjuvant treatments developed to reduce the risk of relapse and mortality underwent substantial changes, leading to a need to re-examine the relevance of new findings from recently treated patients.

We analysed the pathological information obtained at surgery and not at time of diagnosis, so we excluded patients who achieved pCR and those who were not amenable to surgery. However, through the analyses of the histopathological features at final surgery, we aimed to identify a subset of patients with high risk of early treatment failure. This study provides insight to treatments and prognoses of patients with LABC, because it is based on a relatively large population of patients and on data collected in a relatively short period of time, thus allowing consideration of modern treatment procedures. The pathologists, surgeons and medical oncologists used consistent approaches during the years of reference. The results provide substantial additional evidence to support the hypothesis that steroid hormone receptor status defines distinct biological entities requiring a differentiated approach to treatment and clinical trial investigation in LABC. Limited data are available on the relationship between number of cells expressing hormone receptors and outcome in large operable tumours. This might be explained by the heterogeneity of the assays and especially of cutoffs used in the various studies and by the fact that initial biopsy upon which diagnosis and biological characteristics were define was small in size not allowing the full confidence in declaring the findings as representative for the entire neoplasia. A potential role for steroid hormone receptors in defining prognosis and responsiveness to cytotoxic treatment has been reported (Lippman and Allegra, 1980; Colleoni *et al*, 2001, 2004; Kaufmann *et al*, 2007; Torrisi *et al*, 2007). In most of the trials mentioned above, however, analyses were commonly performed based on a so-called ‘receptor-negative grouping’, which combines receptor-negative disease with those expressing low receptor levels. In the present study, we demonstrated a higher risk of early relapse for endocrine nonresponsive disease (defined as having no steroid hormone receptor staining at all). In particular, the results of the present study indicate that RFS at 2 years after primary therapy and surgery is significantly worse in the cohort of patients with ER- and PgR-negative tumours compared with ER-positive breast cancer, either in the NIBC or in IBC group.

Retrospective studies that compared outcome of patients with locally advanced breast of inflammatory and noninflammatory types suggested that IBC is clearly associated with a worse prognosis. In particular, the large experience on factors predicting outcome in LABC was recently published by investigators from the MD Anderson Cancer Center (MDACC; Cristofanilli *et al*, 2007). Patients with IBC had decreased RFS compared with NIBC tumours (5-year RFS, 33.1 *vs* 44.7%, *P*=0.03) and worse OS (5-year OS, 38.5 *vs* 52.1%, *P*<0.01), respectively. Looking at the two cohorts without focusing on endocrine responsiveness, we did not observe a significant difference in RFS (*P*=0.72), DFS (*P*=0.98) and OS (*P*=0.35) between patients with IBC and cT4 NIBC. In particular, DFS at 2 years was 57 and 57%, respectively, for the two cohorts of patients.

As shown in Table 1, about 60% of the patients had endocrine responsive disease. In these patients, a moderate but continuous increased risk of relapse persisting over time can be hypothesised indicating a need to reanalyse the data after a prolonged follow-up.

Ki-67 is an antigen present in all phases of the cell cycle except G_0_ (Greene *et al*, 2002). It is a measure of tumour proliferation that has been correlated with outcome in several studies (Sahin *et al*, 1991; Domagala *et al*, 1996; Pietiläinen *et al*, 1996; Clahsen *et al*, 1999; Mandard *et al*, 2000). It has also been suggested that high Ki-67 LI may be predictive of responsiveness to neoadjuvant chemotherapy (Jansen *et al*, 1998; Chang *et al*, 2000; Archer *et al*, 2003; Trihia *et al*, 2003). The results of the present study indicate that measures of tumour cell proliferation could potentially identify patients who might require further therapy (adjuvant chemotherapy as well as endocrine therapy) after surgery in LABC. In fact, we observed worse RFS in patients with higher staining for Ki-67 compared with a lower degree of staining. However, Ki-67 expression was measured at time of surgery (i.e., after neoadjuvant chemotherapy) and the value at time of diagnosis is not available for all patients. So, we did not analyse the potential role of modification of Ki-67 expression before, during and after neoadjuvant chemotherapy (Bottini *et al*, 2001; Burcombe *et al*, 2006). Anyway our findings are consistent with the conclusions of a recent meta-analysis on the prognostic role of Ki-67 LI in more than 12 000 patients with early breast cancer (de Azambuja *et al*, 2007).

Moreover, our analyses show that the presence of PVI significantly correlate with high risk of early recurrence. The prognostic role of the extent of PVI in operable breast cancer was recently reported (Colleoni *et al*, 2007). Therefore, the presence of vascular invasion should be considered in the therapeutic algorithm to properly select targeted adjuvant treatment.

Limited data are available on the relationship between HER2 overexpression and outcome in LABC. In two large studies focusing on preoperative therapy DFS was significantly worse for those patients whose tumours overexpressed HER2, as compared with those with HER2-negative tumours (Gregory *et al*, 2000; Guarneri *et al*, 2006). Results in LABC are conflicting and do not support an independent prognostic role for HER2 status (Tulbah *et al*, 2002), as observed in the current study. We reached the same result about HER2 prognostic role also when we included in the analysis patients who received trastuzumab therapy.

Inclusion of targeted anti-HER2 therapy in the primary systemic treatment programme for selected patients with HER2-positive LABC might improve treatment outcome for these patients. Results from two randomised trials comparing primary chemotherapy including taxanes and anthracyclines plus/minus trastuzumab indicated that the clinical course of HER2-positive breast cancer can be modified by the preoperative use of targeted therapies (Buzdar *et al*, 2007; Gianni *et al*, 2007).

In conclusion, this study confirms the value of prognostic parameters assessed at final surgery, including ER and PgR expression, Ki-67 expression and presence of vascular invasion. However, these results could be helpful to identify a subgroup of patients with very high risk of early relapse for which we need improved adjuvant therapy. Database analyses with longer follow-up (especially for patients with endocrine responsive disease) or prospective trials will be useful to confirm the value and limitations of these factors in patients with LABC. Future adjuvant treatments should anyhow consider tailored interventions to be added to conventional combined preoperative therapy, because these are likely to improve the clinical outcome of patients with LABC, who otherwise will continue to have a dire prognosis. A treatment for these cohorts of patients within the context of clinical research programmes (i.e., prospectively defined tailored treatments and long-term mandatory follow-up) should be viewed as standard of care for a faster progress in the field.

## Figures and Tables

**Table 1:** Patient and disease characteristics at surgery

	**NIBC, *N* (% collected)**	**IBC, *N* (% collected)**	***P* a**
Total no.	141	107	
*Year of surgery*			
1999–2001	65 (46.1)	16 (14.9)	
2002–2003	35 (24.8)	35 (32.7)	<0.001
2004–2006	41 (29.1)	56 (52.3)	
			
*Age (years)*			
<35	5 (3.5)	4 (3.7)	
35–49	53 ()37.6	34 (31.8)	
50–59	58 (41.1)	34 (31.8)	0.07
60–69	19 (13.5)	30 (28.0)	
70+	6 (4.3)	5 (4.7)	
			
*Histologic type*			
IDC	126 (90.6)	88 (86.3)	
ILC	6 (4.3)	5 (4.9)	0.44
Others	7 (5.1)	9 (8.8)	
NA	2	5	
			
*Lymph node involvement*			
None	11 (7.9)	12 (11.2)	
1–3	30 (21.4)	18 (16.8)	0.51
4+	99 (70.7)	77 (72.0)	
Unknown	1	—	
			
*Ki-67*			
<20%	67 (51.1)	41 (42.3)	0.22
⩾20%	64 (48.9)	56 (57.7)	
NA	2	5	
Unknown	8	5	
			
*ER/PgR status*			
Positive^b^	39 (28.7)	17 (17.0)	
Low^c^	52 (38.2)	40 (40.0)	0.08
Negative	45 (33.1)	43 (43.0)	
NA	2	5	
Unknown	3	2	
			
*HER2 status*			
Overexpressed	35 (25.4)	23 (22.5)	
Not overexpressed	103 (74.6)	79 (77.5)	0.65
NA	2	5	
Unknown	1	—	
			
*Peritumoral vascular invasion*			
Absent	70 (50.4)	42 (41.2)	
Present	69 (49.6)	60 (58.8)	0.30
NA	2	5	

ER=oestrogen receptors; IBC=inflammatory breast cancer; IDC=invasive ductal carcinoma; ILC=invasive lobular carcinoma; NA=not available in patients with pathological complete response; NIBC=noninflammatory breast cancer; PgR=progesterone receptors.

a Unknowns and NA were excluded.

b Both ER and PgR ⩾10%.

c ER or PgR between 1 and 9%.

**Table 2:** Primary treatment characteristics

	**NIBC, *N* (% collected)**	**IBC, *N* (% collected)**	***P***
*Regimen*			
Anthracyclines	74 (52.5)	77 (72.0)	
Anthracyclines and taxanes	50 (35.4)	24 (22.4)	
Others	17 (12.1)	6 (5.6)	0.007
			
*Infusional* a			
Infusional therapy	54 (38.3)	61 (57.0)	
No infusional therapy	87 (61.7)	46 (43.0)	0.005

IBC=inflammatory breast cancer; NIBC=noninflammatory breast cancer.

a Regimen including continuous infusional fluorouracil.

**Table 3:** Univariate and multivariate Cox's models for recurrence-free survival

	**Univariate HR (95% CI)**	***P***	**Multivariate HR (95% CI)^a^**	***P***
*cT*				
4d (IBC) *vs* 4a–c (NIBC)	1.08 (0.72–1.62)	0.72	0.73 (0.46–1.15)	0.17
				
*Year of surgery*				
2003–2006 *vs* 1999–2002	0.96 (0.74–1.24)	0.77	—	—
				
*Age*				
⩾50 *vs* <50	0.91 (0.61–1.36)	0.64	—	—
				
*Lymph node involvement*				
1–3 *vs* none<4	1.26 (0.46–3.44)		—	
⩾4 *vs* none<4	1.58 (0.64–3.90)	0.45	—	—
				
*ER/PgR status*				
Low *vs* positive	1.00 (0.54–1.83)		1.12 (0.59–2.13)	
Negative *vs* positive	2.49 (1.42–4.35)	<0.001	2.47 (1.33–4.59)	<0.001
				
*HER2 status*				
Overexpressed *vs* not overexpressed	1.30 (0.84–2.02)	0.24	—	—
				
*Ki-67 LI*				
⩾20 *vs* <20%	3.50 (2.17–5.65)	<0.001	2.69 (1.61–4.50)	<0.001
				
*Peritumoral vascular invasion*				
Present *vs* absent	1.75 (1.16–2.65)	0.008	2.27 (1.42–3.62)	<0.001
				
*Neoadjuvant response*				
No response *vs* complete/partial	1.49 (0.99–2.22)	0.054	1.07 (0.69–1.68)^b^	0.76
				
*Regimen*				
Anthracyclines and taxanes *vs* anthracyclines or others	1.20 (0.77–1.86)^b^	0.43	—	—

IBC=inflammatory breast cancer; NIBC=noninflammatory breast cancer.

a The seven patients with complete pathological response were not considered, as the biological variable at surgery was not available.

b Estimate relative to partial response *vs* no response.
